# Gut microbiota and its metabolites in non-small cell lung cancer and brain metastasis: from alteration to potential microbial markers and drug targets

**DOI:** 10.3389/fcimb.2023.1211855

**Published:** 2024-01-18

**Authors:** Haixiao Jiang, Wei Zeng, Xiaoli Zhang, Yuping Li, Yilun Wang, Aijun Peng, Demao Cao

**Affiliations:** ^1^ Department of Neurosurgery, The Affiliated Hospital of Yangzhou University, Yangzhou, China; ^2^ Department of Neurosurgery, Yancheng First Hospital, Affiliated Hospital of Nanjing University Medical School, Yancheng, China; ^3^ Department of Medical Imaging, The Affiliated Hospital of Yangzhou University, Yangzhou, China; ^4^ Department of Neurosurgery, Clinical Medical College of Yangzhou University, Yangzhou, China; ^5^ Department of Thoracic Surgery, Clinical Medical College of Yangzhou University, Yangzhou, China

**Keywords:** gut microbiota, lung cancer, brain metastasis, SCFAs, gut-lung axis

## Abstract

**Background:**

The elevated mortality rate associated with non–small-cell lung cancer (NSCLC) is a well-established global concern. Considerable attention has been directed toward exploring the association between gut microbiota and various malignant tumors. We herein investigated the associations between the intestinal microbiome and its metabolites, particularly short-chain fatty acids (SCFAs), in patients with NSCLC at different stages, including early and brain metastasis (BM) stages. The findings aim to offer a fresh perspective on the diagnosis and management of NSCLC.

**Methods:**

Fecal samples were collected from 115 participants, comprising healthy controls (n = 35) and patients with treatment-naive NSCLC at the early stage (ELC, n = 40) and the BM stage (n = 40). Characterization of the intestinal microbiome and fecal SCFA levels was performed using 16S rRNA gene sequencing and gas chromatography.

**Results:**

The microbial diversity in patients with NSCLC was found to be less abundant and uniform, particularly in the BM stage. Significant alterations in the community structure of the gut microbiota were observed in patients with NSCLC, with an increase in pathogens in Fusobacteria and Proteobacteria and a decrease in SCFA-producing bacteria in Firmicutes and Actinobacteria, particularly in the BM stage. Meanwhile, microbial communities displayed intricate associations in patients with NSCLC. A biomarker panel (*Faecalibacterium*, *Bifidobacterium*, *Butyricicoccus*, *Klebsiella*, *Streptococcus*, and *Blautia*) successfully distinguished patients in the ELC and BM stages from healthy controls (area under the curve: 0.884). The overall concentration of fecal SCFAs was significantly lower in patients with BM compared to patients with ELC and healthy controls. Subgroup analysis of acetate and butyrate yielded similar results. Moreover, multiple disrupted pathways in the NSCLC group were identified using the Kyoto Encyclopedia of Genes and Genomes annotation, including lipid metabolism and genetic information processing, specifically in the BM stage.

**Conclusion:**

Compared with healthy controls, distinct host-microbe interactions were evident in different phases of patients with NSCLC. Furthermore, specific forms of the gut microbiome and SCFAs may serve as valuable biomarkers and therapeutic targets in the diagnosis and treatment of NSCLC.

## Introduction

Lung cancer (LC) is renowned for its high malignancy and mortality rates. Globally, the average annual incidence of LC is approximately 31.5/100,000, with the incidence in China exceeding 40/100,000 (Sung et al., 2021). Non–small-cell lung cancer (NSCLC) constitutes over 85% of lung cancers (Siegel et al., 2023). Despite the development of various medications for NSCLC, few can effectively prevent distant metastasis, particularly brain metastasis (BM). Brain metastasis is well-known for its high invasiveness and poor prognosis (Siegel et al., 2023). The heredity of LC susceptibility poses a significant obstacle, with genetic researchers identifying poor heredity as a key challenge. Although studies have highlighted the importance of inherited familial hazards and somatic mutations (Li et al., 2018; Shen et al., 2019), heritability is only partially explained by genetic effects, and results depend on unique responders.

Conversely, non-genetic factors, including environmental factors, behaviors, and microbiota, are considered essential LC etiological factors. Emerging research suggests that microorganisms may significantly impact LC progression (Jin et al., 2019; Zhuang et al., 2019. Commensal microorganisms, as found by Jin et al. (Jin et al., 2019), stimulate myeloid cells to produce Myd88-dependent IL-1β and IL-23, inducing the activation and proliferation of Vγ6+Vδ1+γδ T cells, ultimately advancing tumor cell proliferation and inflammation. Another study by Lin et al. (Liu et al., 2023) revealed that *Aspergillus sydowii* residing in lung adenocarcinoma promotes tumor progression by suppressing the activity of cytotoxic T lymphocytes. While many LC microorganism studies focus on lung microbes (Lee et al., 2016; Cameron et al., 2017), recent research has associated oral microorganisms with LC incidence and progression (Christiani, 2021; Hosgood et al., 2021). Hosgood et al. (Hosgood et al., 2021) found that lower alpha diversity of oral microorganisms suggests a higher risk of lung cancer, potentially linked to fundamental processes such as immunoreaction, metabolism, and genotoxicity (Mao et al., 2018).

The most complex microbiome, the intestinal microbiome, significantly affects immunological homeostasis, metabolic balance, and tissue development (Liu et al., 2021a). The intestinal microbiome can influence gastrointestinal tumors (Yang et al., 2022) and has been associated with various extraintestinal tumors (Zhang et al., 2021; Jiang et al., 2022). Recent studies have explored a possible association between the intestinal microbiome and NSCLC. Vernocchi P (Vernocchi et al., 2020) reported that *Rikenellaceae*, *Oscillospira*, and *Bacteroides plebeians* were enriched in NSCLC. Additionally, Zhang (Zhang et al., 2018) found significant differences in eight predominant genera, including *Escherichia/Shigella*, *Dialister*, *Enterobacter*, *Kluyvera*, *Fusobacterium*, *Veillonella*, and *Bacteroides*, between patients with NSCLC and healthy controls (HCs). Emerging evidence also suggests that specific changes in individual species, such as *Prevotella copri* and *Bifidobacterium longum*, and the general diversity of the intestinal microbiome are linked to the response to immunotherapy (Jin et al., 2019). Meanwhile, the intestinal microbiome and its metabolite, particularly short-chain fatty acids (SCFAs), may contribute to NSCLC development. SCFAs have been shown to modulate inflammatory and immunological responses by inhibiting histone deacetylases and activating G-protein-coupled receptors (GPRs). A deficiency in SCFAs may contribute to the progression of various tumors, including distant metastases (Hou et al., 2022). In conclusion, increasing data suggest that the intestinal microbiome and its metabolites significantly affect tumor progression by modulating immunoreaction and inflammation, as well as interacting directly with targeted drugs (Dohlman et al., 2022; Huang et al., 2022).

Despite these substantial achievements, several questions persist in these research endeavors. First, many studies have overlooked the dynamic microbial shifts during the various stages of NSCLC. Second, the variations associated with BM of NSCLC have not yet been adequately described. Thirdly, research on the underlying association between NSCLC and variations in microbiota and its metabolite, particularly SCFAs, is currently lacking. The primary objective of this study was to address these challenges by investigating gut microbiota and SCFAs in patients with NSCLC at various stages (early stage and BM stage). Additionally, the aim was to establish a microbial biomarker panel capable of distinguishing patients with NSCLC, specifically those with BM, from HCs. This approach could enhance our understanding of the progression and metastasis of lung tumors, leading to the identification of novel therapeutic targets and robust microbial biomarkers for clinical management. In the future, fecal microbial transplant and the consumption of SCFAs may emerge as fundamental adjuvant therapeutic techniques for NSCLC and even BM.

## Materials and methods

### Sample collection and research design

The written informed consent was signed by 115 participants, consisting of 80 patients with treatment-naive NSCLC at various stages (i.e., WHO I–IV, defined in accordance with the 8th American Joint Committee on Cancer guidelines, with BM representing stage IV), and 35 HCs. From June 2021 to February 2023, all patients admitted to The Affiliated Hospital of Yangzhou University underwent medical imaging and stool examinations.

The subjects were all Han Chinese, from the same region, and consumed identical meals. Subjects were excluded if they met the following requirements: those who were too young or old (<18 or >80 years); those with a family genetic history of any gastrointestinal diseases or LC; those with behavior changes (particularly eating behavior patterns); those who took antibiotics or probiotics 2 months before the collection of the fecal specimen; those who had received any medical treatment such as pharmacological treatment and surgery before collection; and those with abnormal intestinal habits (constipation or diarrhea). Additionally, we took fecal samples from each subject within 3h after hospitalization using a sterile cotton swab. The swabs were promptly preserved in a specific solution and stored at −20°C for future detection. This study is registered with the Chinese Clinical Trial Register (ChiCTR2100044256) and received approval from The Affiliated Hospital of Yangzhou University’s Ethics Committee.

### DNA extraction

Genomic DNA was extracted using the PowerMax extraction kit (TIANGEN Biotech, China), and then frozen at −80°C. Next, a NanoDrop ND-1000 spectrophotometer (Thermo Fisher Scientific, USA) was utilized to measure both the quantity and quality of DNA.

### 16S rRNA amplicon pyrosequencing

The V4 region of the microorganism 16S rRNA gene was chosen for PCR amplification (515F:5′-GTGCCAGCMGCCGCGGTAA-3′ and 806R:5′-GGACTACHVGGGTWTCTAAT-3′ served as the forward and reverse primer, respectively). The sample-specific exact 6-bp sequence was synthesized into TrueSeq adaptors for multiplex sequencing. The PCR amplification system comprised forward and reverse primers (both 3 μL), Phusion High-Fidelity PCR Master Mix (25 μL), DNA Template (10 μL), ddH2O (6 μL), and DMSO (3 μL). The thermal cycling process included pre-denaturation at 98 degrees Celsius for 30 s, followed by 25 cycles of denaturation (98 degrees Celsius, 15 s), annealing (58 degrees Celsius, 15 s), extension (72 degrees Celsius, 15 s), and final elongation (72 degrees Celsius, 60 s). After purification with AMPure XP Beads (Omega Bio-Tek, USA), we measured PCR amplicons with the PicoGreen dsDNA Assay Kit (Invitrogen, USA) and then pooled amplicons in equimolar proportions. The Illumina HiSeq4000 platform was employed for pair-end 2 × 150 bp sequencing.

### Analysis of the concentration of fecal SCFAs

For the quantitative study of fecal levels of SCFAs, a gas chromatograph (Waters Corporation, Milford, USA) in conjunction with a flame ionization detector was utilized. Fecal samples were prepared according to a validated protocol (Ubachs et al., 2021). A vacuum dryer (Memmert, Schwabach, Germany) was employed to dry 500 mg of frozen feces for 5 h, and the fecal levels of SCFAs were then corrected by the dry weight.

### Sequence analysis

First, we assigned raw sequencing reads to respective samples using sample-specific barcodes. Vsearch (v2.22.1.) was employed to aggregate paired-end reads. The amplicon sequence variant (ASV) picking utilized Vsearch, involving dereplication, quality control, denoising sequences with the UNOISE2 algorithm (Edgar, 2016), Chimera removal (Rognes et al., 2016), and mapping to ASVs with a 100% similarity threshold. To minimize differences in sequencing depth across samples, normalization was performed on the ASV sequence counts, ensuring the sum of ASV sequences in each sample was consistent. Normalized values were set to 1, representing relative abundances. A representative sequence was selected from each ASV based on default parameters. Rep-seqs and ASV table files were imported into QIIME2 (QIIME2-2022.2) (Bolyen et al., 2019). ASVs with less than 0.001% of total sequences across all samples were discarded by QIIME2. Taxonomic identifications were assigned using QIIME2’s weighted taxonomic classifiers, and the taxonomy generated was collapsed (Levels phylum to genus) using the “qiime taxa collapse” command.

### Bioinformatics and clinical data analysis

The α-diversity indexes (Shannon, Simpson, Chao) were used to assess the bacterial ecosystem’s complexity using the QIIME2-diversity core-metrics phylogenetic tool (Rai et al., 2021). The beta diversity analysis was computed using principal coordinate analysis and nonmetric multidimensional scaling (Shi et al., 2020). Statistical significance was assessed using the analysis of similarities method (Salaheen et al., 2020). Taxa abundances among groups at various levels (phylum to genus) were compared. Linear discriminant analysis effect size was used to analyze the influence of differentially abundant taxa (Yu et al., 2022). Diagnostic model accuracy was evaluated through receiver operating characteristic (ROC) curves and the area under the curve. Pathway enrichment was examined using Kyoto Encyclopedia of Genes and Genomes (KEGG) annotations (Kanehisa et al., 2021). The Statistical Analysis of Metagenomic Profiles (STAMP, v2.1.3.) software package was used for further analysis, and high-level phenotypes in the microbiome were measured using the BugBase tool (Wang et al., 2020). The Sequence data analyses were primarily conducted using R packages (v4.1.0) and QIIME2, while clinical data analyses were performed with SPSS (version 26.0) and R software (v4.1.0). The differences between continuous variables were calculated using the one-way analysis of variance or Kruskal–Wallis test for three groups (variables were normally distributed) and the Mann-Whitney U-test or an independent t-test for two groups (variables were not normally distributed). Fisher’s exact or chi-square tests were used to examine categorical variable differences, considering it significant only when the false discovery rate (FDR)-corrected *p*-value was <0.05.

## Results

### Clinical characteristics of participants

In this study, 115 participants were enlisted, comprising 80 patients with NSCLC without prior therapy and 35 HCs. The terms “ELC,” “BM,” and “HC” were used to refer to early-stage NSCLC (stages I–III), brain metastasis, and healthy controls, respectively. Demographic characteristics were collected to ensure baseline data equally matched among the groups (**Table 1**).

**Table 1:** The baseline characteristics of all study participants.

	ELC	BM	HC	HC vs LC	ELC vs. BM
	(n=40)	(n=40)	(n=35)	*p* value	*p* value
**Age (Mean ± SD)**	56.25 ± 11.85	57.83 ± 9.22	54.46 ± 8.98	0.211	0.509
**Gender**				0.725	0.478
Male	25 (62.5%)	28 (70%)	22 (62.9%)		
Female	15 (37.5%)	12 (30%)	13 (37.1%)		
**BMI**	22.72 ± 2.14	22.10 ± 2.24	22.31 ± 2.27	0.913	0.202
**Smoking**				0.447	0.251
Absence	18 (45.0%)	15 (37.5%)	18 (51.4%)		
Presence	22 (55.0%)	25 (62.5%)	17 (48.6%)		
**Drinking**				0.856	0.428
Never	30 (75%)	27 (67.5%)	25 (71.4%)		
<1 standard drink per day	4 (10%)	3 (7.5%)	4 (11.4%)		
≥1 standard drink per day	6 (15%)	10 (25%)	6 (17.1%)		
**Hypertension**				0.785	0.672
Negative	31 (77.5%)	28 (70%)	26 (74.3%)		
Positive	9 (22.5%)	12 (30%)	9 (25.7%)		
**Diabetes**				0.142	0.982
Negative	36 (90%)	35 (87.5%)	34 (97.1%)		
Positive	4 (10%)	5 (12.5%)	1 (2.9%)		
**Excrement regularity**				0.952	0.831
Yes	37 (92.5%)	35 (87.5%)	31 (88.6%)		
No	3 (7.5%)	5 (12.5%)	4 (11.4%)		
**Cancer pathology**				–	–
Adenocarcinoma	25(62.5%)	28(70%)	–		
Squamous cell carcinoma	12(30%)	8(20%)	–		
Other types	3(7.5%)	4(10%)	–		
**TNM stage**				–	–
Stage I	14 (35%)	0 (0%)	–		
Stage II	9 (22.5%)	0 (0%)	–		
Stage III	17(42.5%)	0 (0%)	–		
Stage IV	(0%)	40(100%)	–		

HC, healthy group; ELC, an early stage of non-small cell lung cancer; BM, brain metastasis; SD, standard deviation; TNM, tumor-node-metastasis.

### Changes in gut microbiota composition in patients with ELC and BM

**Figure 1:**
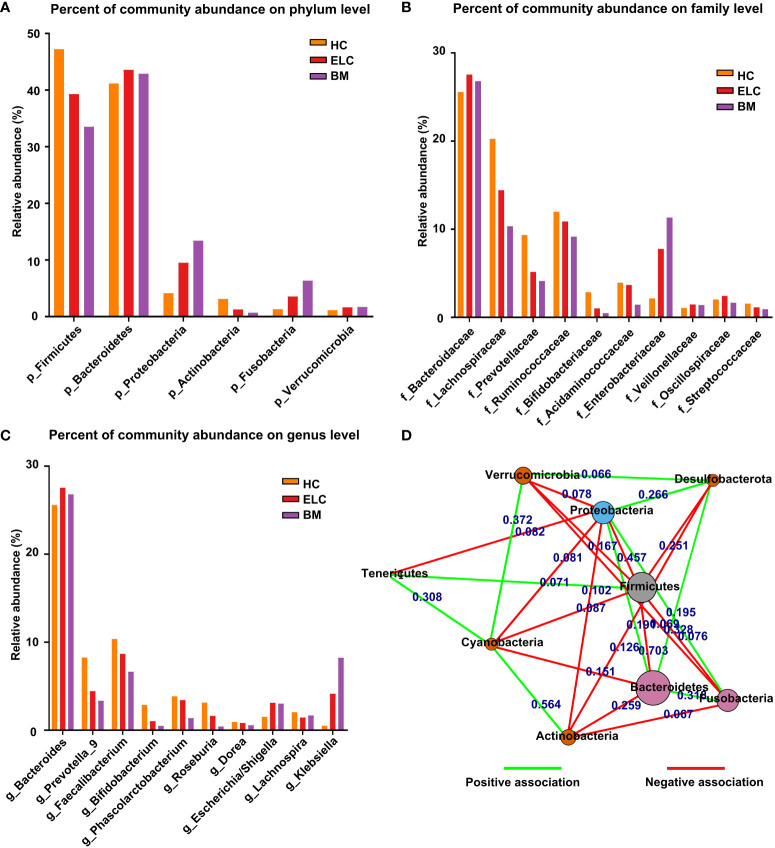
The proportion of dominant phylum-level intestinal flora in each group **(A)**. The relationships between different phylum-level microbiota in NSCLC patients **(D)**. Each node represents a phylum type, and the length of the lines between them represents the strength of the correlation (the shorter the line, the stronger the correlation). The red color represents negative relationships, while the green color indicates positive ones. The dominant microbial family **(B)** and genera **(C)** among the groups. ELC, the early stage of non-small cell lung cancer; BM, brain metastasis; HC, healthy group.

The relative taxon abundance of the three groups was compared to examine the characteristics of gut microbiota in patients with ELC and BM, revealing an unexpected variance. Firmicutes, Bacteroidetes, Proteobacteria, Actinobacteria, and Fusobacteria constituted 96.94% of all microorganisms in the HC group. The top five phyla in the ELC and BM groups were Bacteroidetes, Firmicutes, Proteobacteria, Fusobacteria, and Verrucomicrobia, accounting for 97.57% and 97.86% of their respective groups. Additionally, a significant increase in Proteobacteria (4.14% vs. 9.51% vs. 13.41%, *p* = 0.035, FDR-corrected *p =* 0.025) and Fusobacteria (1.30% vs. 3.56% vs. 6.34%, *p* = 0.018, FDR-corrected *p =* 0.025) was observed in patients with NSCLC, particularly patients with BM. However, Firmicutes (47.22% vs. 39.30% vs. 33.52%, *p* = 0.042, FDR-corrected *p =* 0.025) and Actinobacteria (3.12% vs. 1.27% vs. 0.69%, *p* = 0.023, FDR-corrected *p =* 0.025) showed the opposite trend. Meanwhile, a reduced ratio of Firmicutes to Bacteroidetes (F/B) was revealed (*p* = 0.029, FDR-corrected *p =* 0.025) (**Figure 1A**; **Supplementary Table 1**).

*Bacteroidaceae*, *Lachnospiraceae*, *Ruminococcaceae*, *Prevotellaceae*, and *Acidaminococcaceae* exhibited the highest relative abundance among microbiome families in the HC group. In contrast, the ELC group demonstrated notable abundance in *Bacteroidaceae*, *Lachnospiraceae*, *Ruminococcaceae*, *Enterobacteriaceae*, and *Prevotellaceae*. The BM group displayed substantial abundance in *Bacteroidaceae*, *Enterobacteriaceae*, *Lachnospiraceae*, *Ruminococcaceae*, and *Prevotellaceae*, ranking them as the five most relatively large microbiomes (**Figure 1B**; **Supplementary Table 1**).

In this study, the classification of genera revealed a more intricate pattern. The HC group exhibited the highest relative abundance in *Bacteroides*, *Faecalibacterium*, *Prevotella_9*, *Phascolarctobacterium*, and *Roseburia* as the top five microbiomes. Conversely, the ELC group displayed prevalence in *Bacteroides*, *Faecalibacterium*, *Prevotella_9*, *Klebsiella*, and *Phascolarctobacterium*. In contrast, the BM group demonstrated predominance in *Bacteroides*, *Klebsiella*, *Faecalibacterium*, *Prevotella_9*, and *Escherichia/Shigella* as the five most pervasive microbiomes (**Figure 1C**; **Supplementary Table 1**).

### Complex associations within various microbial communities

Correlation analysis was performed to investigate the underlying associations within various microbial communities in patients with NSCLC. Furthermore, this study identified 15 negative and 10 favorable associations between the 9 phyla in patients with NSCLC. Firmicutes were negatively associated with Bacteroidetes, Fusobacteria, and Proteobacteria. Proteobacteria, whereas, was negatively associated with Actinobacteria and Verrucomicrobia and positively associated with Bacteroidetes and Fusobacteria (**Figure 1D**).

### Decreased bacterial diversity in the gut microbiota of patients with NSCLC

**Figure 2:**
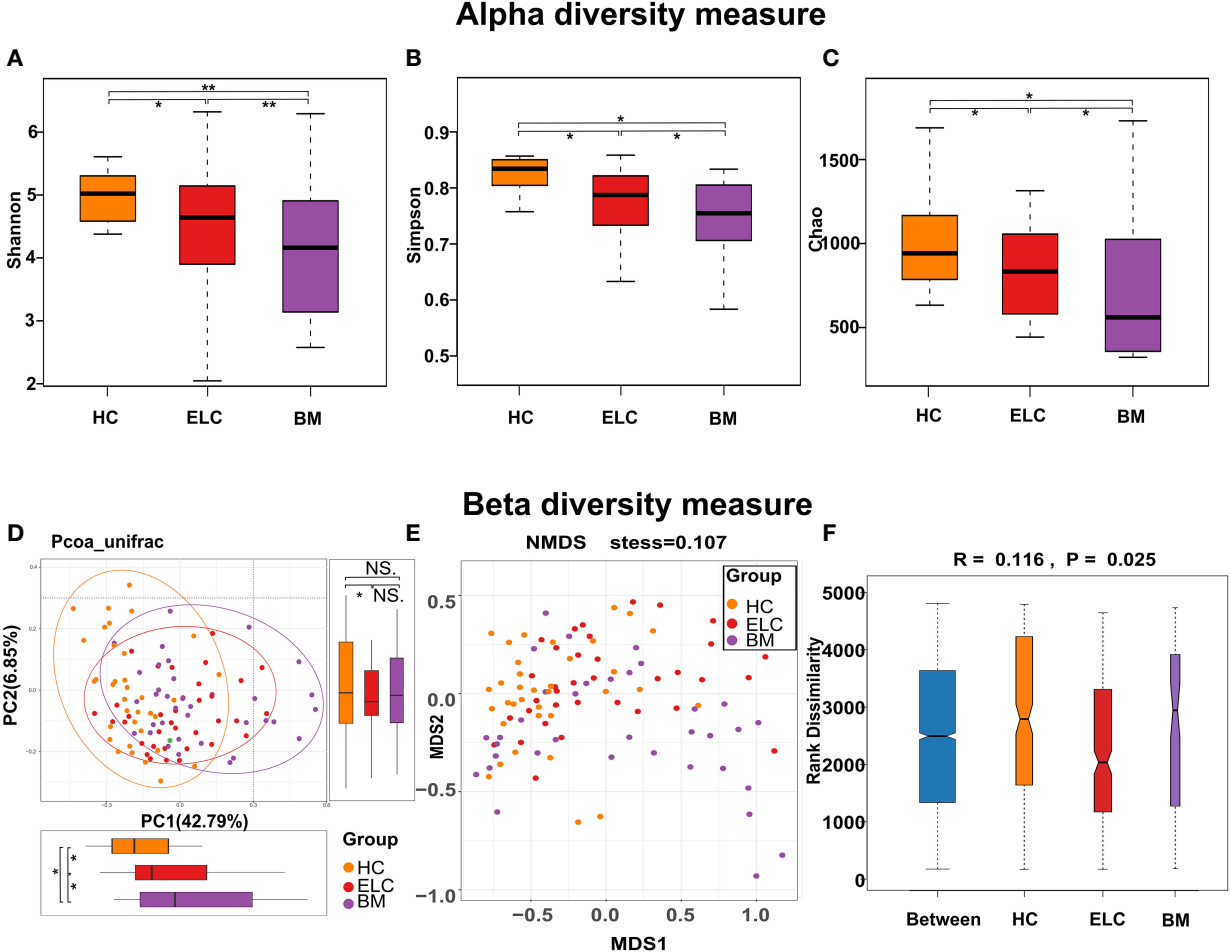
To evaluate the differences in alpha diversity among groups, the Shannon **(A)**, Simpson **(B)**, and Chao indices **(C)** were calculated. Microbial beta diversity of the three groups was calculated by the Principal to Coordinate Analysis (PCoA) based on the unweighted UniFrac distances **(D)** and Nonmetric Multidimensional Scaling based on the Bray-Curtis distances **(E)**. The distance between spots indicates the microbial structural similarity between samples, with each spot representing one sample. The Analysis of Similarities (ANOSIM) among the three groups **(F)** was calculated to compare the differences between the inter-group and intra-group. * 0.01 < *p* < 0.05; ** *p* < 0.01; ELC, the early stage of non-small cell lung cancer; BM, brain metastasis; HC, healthy group.

The alpha diversity of each group was assessed to determine whether the microbial community structure had changed. The results indicated that the alpha diversity of the intestinal microbiome in patients with NSCLC was lower than that in HCs, especially in the BM group. This was evident in the indices of Shannon (HC vs. ELC, FDR-corrected *p* = 0.038; ELC vs. BM, FDR-corrected *p* = 0.0078; HC vs. BM, FDR-corrected *p* = 0.0072, **Figure 2A**), Simpson (HC vs. ELC, FDR-corrected *p* = 0.041; ELC vs. BM, FDR-corrected *p* = 0.041; HC vs. BM, FDR-corrected *p* = 0.0171, **Figure 2B**), and Chao (HC vs. ELC, FDR-corrected *p* = 0.046; ELC vs. BM, FDR-corrected *p* = 0.033; HC vs. BM, FDR-corrected *p* = 0.0147, **Figure 2C**). This suggests that the gut microbiota lose its diversity and evenness with the degree of malignancy. Moreover, Beta diversity analysis, including principal coordinates analysis based on unweighted UniFrac distance (**Figure 2D**) and nonmetric multidimensional scale analysis based on Bray-Curtis distances (stress = 0.107, **Figure 2E**), revealed a significant separation of gut microbiomes among groups. Furthermore, an analysis of similarities was performed, demonstrating a noticeable variation in the organization of the intestinal microbiome among groups (Unweighted UniFrac, *p* = 0.025, *r* = 0.116, **Figure 2F**).

### Potential microbial biomarkers for patients with NSCLC at different stages and HCs

**Figure 3:**
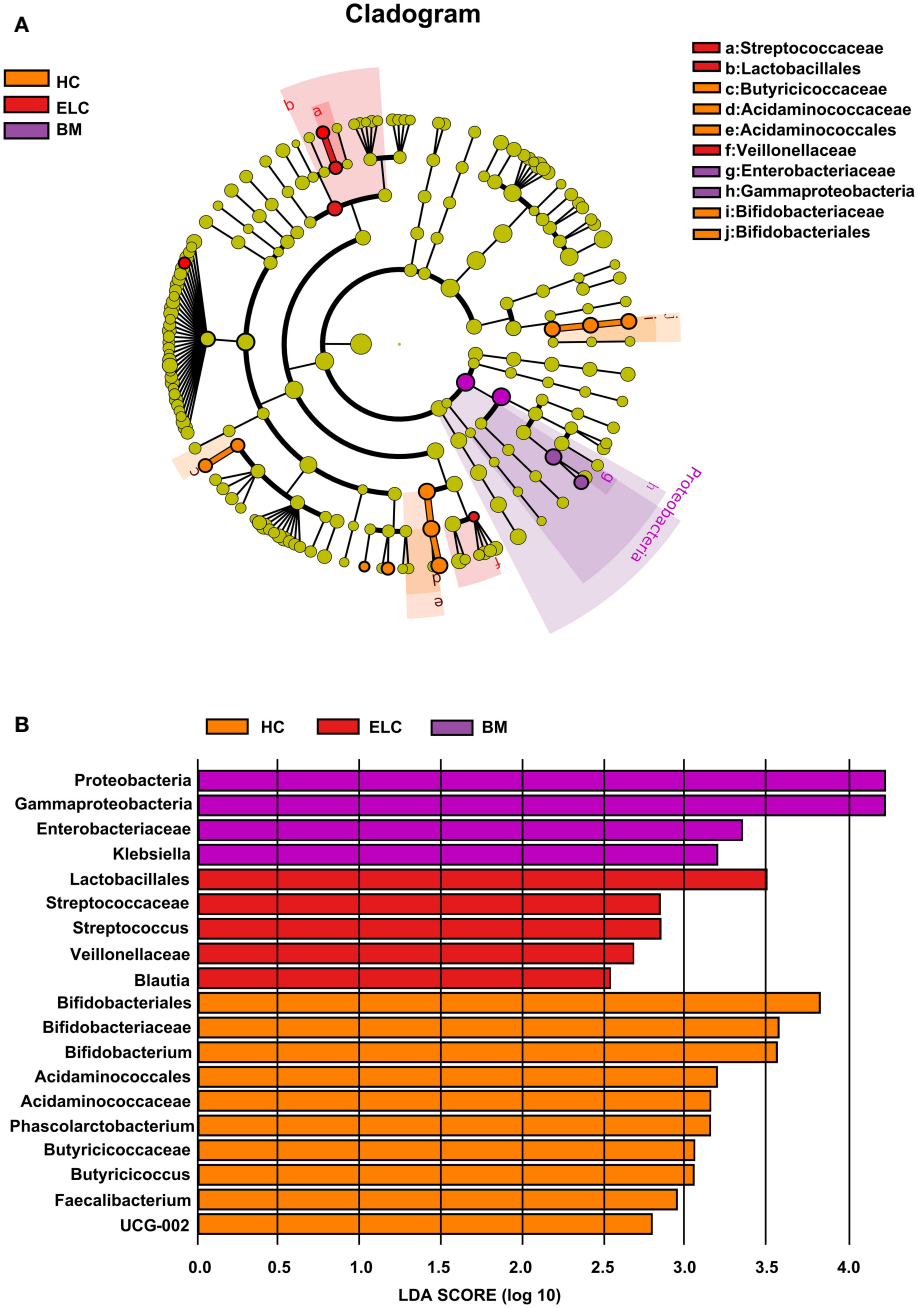
Linear discriminant analysis (LDA) integrated with effect size (LEfSe). Cladogram suggesting the phylogenetic distribution of the gut flora correlated with the healthy group and patients with ELC and BM **(A)**. The inner and outer rings in the radiations indicate the taxonomic level from phylum to genus. The colored node denotes a species classification overrepresented in the corresponding group at that level—the LDA scores of microbial taxa with different abundance **(B)**. Only the taxa with an LDA value >2 are presented. ELC, the early stage of non-small cell lung cancer; BM, brain metastasis; HC, healthy group.

While the composition of the gut microbiota in patients with ELC and BM had changed, discriminant analysis alone could not determine the predominant taxon. Therefore, a linear discriminant analysis effect size was conducted to identify distinct taxa within each group, revealing 19 NSCLC-associated bacterial communities (10 healthy-enriched, 5 ELC-enriched, and 4 BM-enriched, **Figures 3A, B**). Several potential biomarkers for ELC and BM were identified (linear discriminant analysis scores (log10) > 2.5), including *Streptococcus* (an opportunistic pathogen) for ELC and *Enterobacteriaceae* (a typical carcinogenic bacterium) for BM. Meanwhile, numerous SCFA-producing bacteria, including *Bifidobacterium* and *Faecalibacterium*, were significantly enriched in the HCs. These results suggest that the intestinal microbiome may be tightly associated with the malignant degree of NSCLC.

### Microbiome-based signature discriminates patients with ELC and BM from HCs

**Figure 4:**
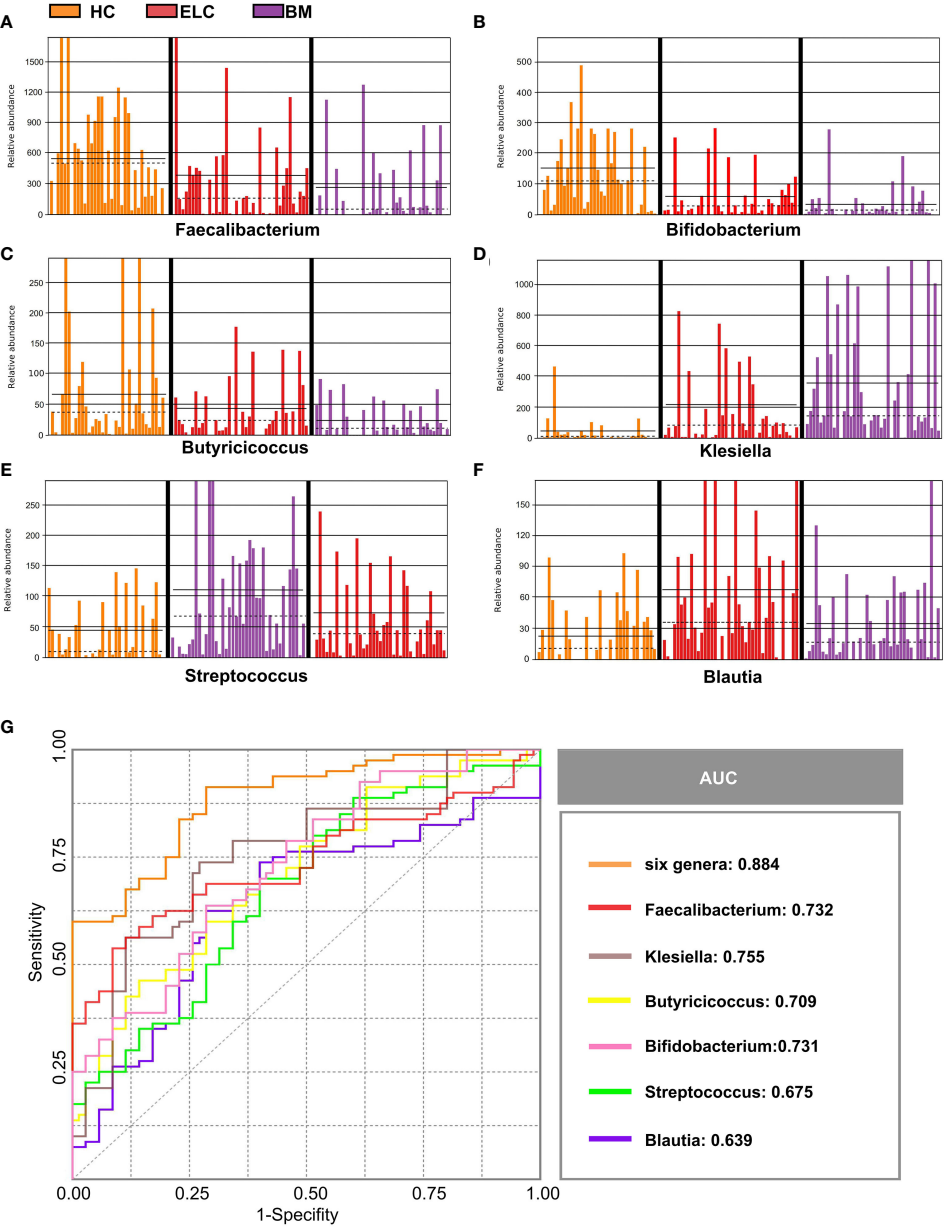
Six abundant genera were identified as biomarkers for NSCLC. The relative abundance of these genera in each sample was displayed, including *Faecalibacterium* **(A)**, *Bifidobacterium* **(B)**, *Butyricicoccus* **(C)**, *Klebsiella* **(D)**, *Streptococcus* **(E)**, and *Blautia* **(F)**. Based on the six genera individually or in combination, the ROC curves were drawn to distinguish patients with ELC and BM from the healthy controls **(G)**. ELC, the early stage of non-small cell lung cancer; BM, brain metastasis; HC, healthy group.

Numerous different species of ELC-associated and BM-associated gut microbiomes were identified in this study. Values were computed using biomarkers from six prevalent genera: *Faecalibacterium*, *Bifidobacterium*, *Butyricicoccus*, *Klebsiella*, *Streptococcus*, and *Blautia*, which exhibited distinctive characteristics and interpersonal variations (**Figures 4A–F**). Each genus was then used as a predictor, and ROC curves with AUC ranging from 0.639 to 0.755 were exhibited in **Figure 4G**. Notably, by combining all six genera, the prediction performance was significantly enhanced (AUC: 0.884). Consequently, these taxa could serve as a biomarker panel to differentiate patients with ELC and BM from HCs.

### Lower fecal SCFA levels in patients with ELC and BM

**Figure 5:**
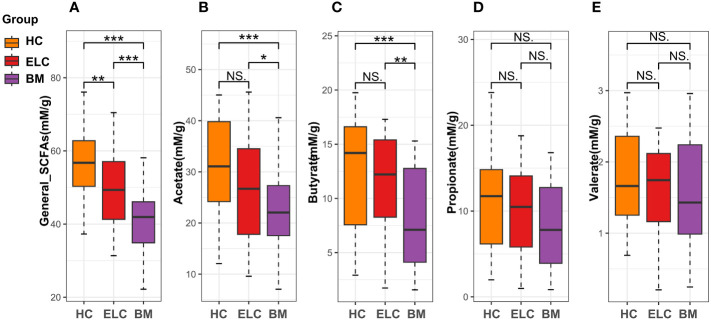
The fecal levels of general short-chain fatty acids (SCFAs, **A**) and acetate **(B)**, butyrate **(C)**, propionate **(D)**, and valerate **(E)**. * 0.01 < *p* < 0.05; ** *p* < 0.01; ELC, the early stage of non-small cell lung cancer; BM, brain metastasis; HC, healthy group.

The general concentration of fecal SCFAs demonstrated a lower tendency in patients with BM (median = 41.93 mM/g, IQR =11.3 mM/g) than in patients with ELC (median = 49.34 mM/g, IQR =15.88 mM/g, *p* < 0.01) and HCs (median = 56.7 mM/g, IQR =14.1 mM/g, *p* < 0.001, **Figure 5A**). This trend was consistent across various SCFAs in BM, with significant decreases in acetate and butyrate concentrations compared with patients with ELC and HCs (**Figures 5B, C**). While propionate and valerate concentrations in BM also decreased, these differences were not statistically significant (**Figures 5D, E**).

### Difference in the functional profile of gut microbiota in patients with ELC and BM and HCs

**Figure 6:**
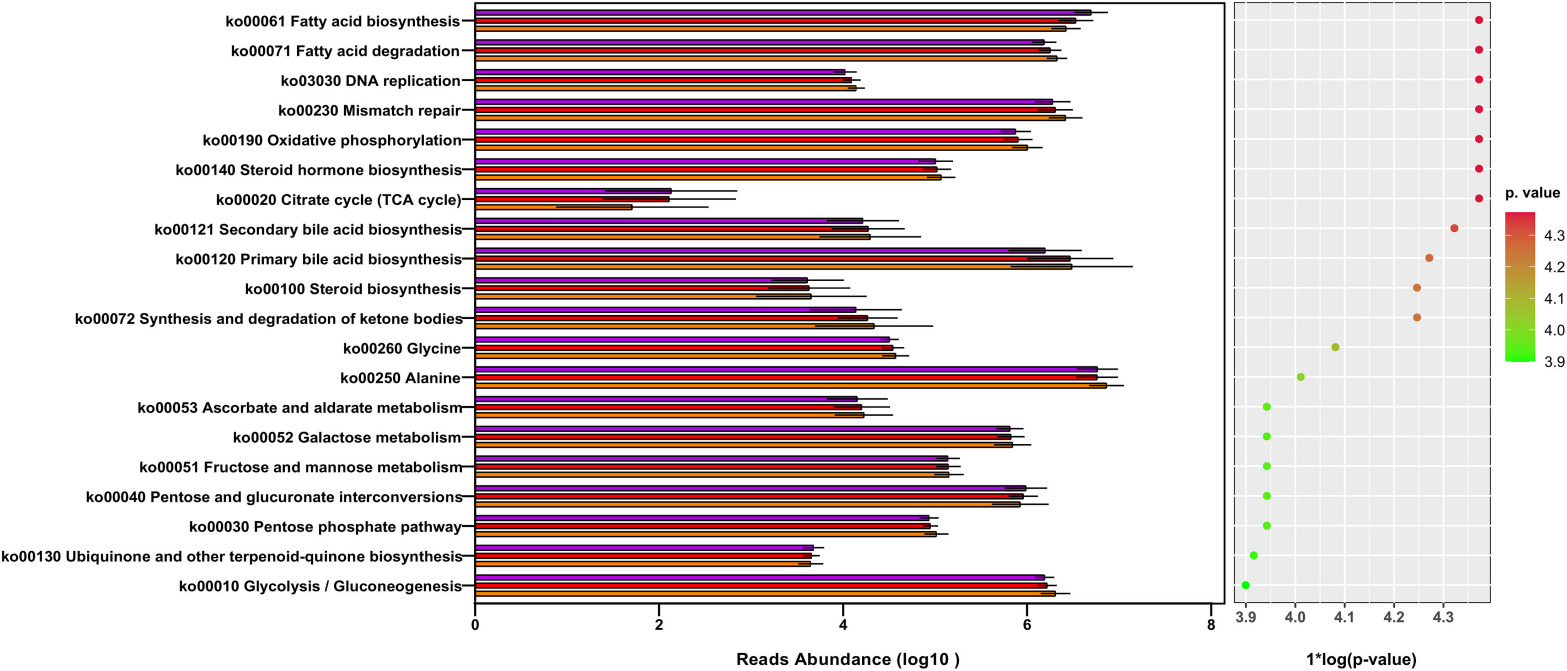
The gut microbiota’s metagenome function was predicted based on a review of the top 20 KEGG pathways. KEGG: Kyoto Encyclopedia of Genes and Genomes. ELC, the early stage of non-small cell lung cancer; BM, brain metastasis; HC, healthy group.

We investigated underlying functional variations in the gut microbiota of patients with ELC and BM using the PICRUSt (Phylogenetic investigation of communities by reconstruction of unobserved states) soft package. The three groups yielded 171 distinct KEGG pathways, with the top 20 modules displayed in **Figure 6**. Although the compositions were somewhat comparable according to the annotation from the KEGG database, the NSCLC group exhibited decreased abundance in most pathways compared with the HCs, particularly in the BM group, including lipid metabolism and genetic information processing. These findings suggest that dysbiosis of the intestinal microbiome is associated with the malignancy grade and enhances the progression of NSCLC.

### Changes in the phenotypic characteristics of the microbiome in NSCLC

**Figure 7:**
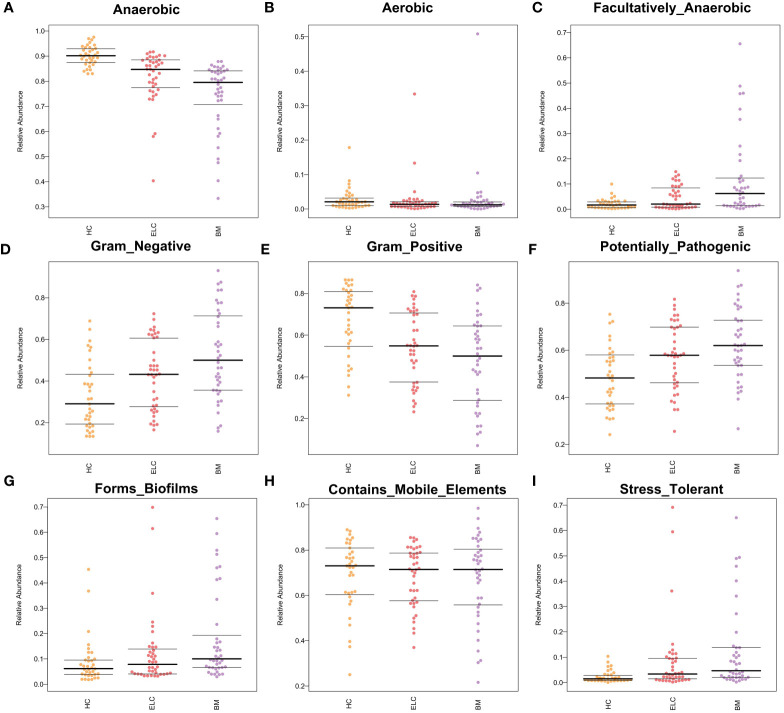
Bugbase software was used to speculate the microbial phenotypic characteristics of Anaerobic **(A)**, Aerobic **(B)**, Facultatively Anaerobic **(C)**, Gram-negative **(D)**, Gram-positive **(E)**, Potentially Pathogenic **(F)**, Forms Biofilms **(G)**, Contains Mobile Elements **(H)**, and Stress tolerance **(I)**. Each spot represents one sample. ELC, the early stage of non-small cell lung cancer; BM, brain metastasis; HC, healthy group.

To determine the potential causes of these associations, we also projected the phenotypic characteristics of the microbiome in all subjects. Bugbase software was implemented, revealing significant increases in facultative anaerobes (FDR-corrected *p* = 0.0043), gram-negative bacteria (FDR-corrected *p* = 0.0040), opportunistic pathogens (FDR-corrected *p* = 0.0040), biofilm formation (FDR-corrected *p* = 0.0042), and oxidative stress tolerance (FDR-corrected *p* = 0.0048) in patients with NSCLC. In contrast, the number of anaerobic bacteria (FDR-corrected *p* = 0.0040) and gram-positive bacteria (FDR-corrected *p* = 0.0040) portrayed different patterns. Additionally, insignificant differences were observed in aerobic bacteria (FDR-corrected *p* = 0.31) and mobile elements (FDR-corrected *p* = 0.33) (**Figures 7A–I**).

## Discussion

As widely acknowledged, the gut microbiota plays a crucial role in maintaining the balance of metabolic function and immunological homeostasis. Microorganism dysbiosis has been implicated in promoting pathological inflammation and potentially contributing to cancer progression (Elkrief et al., 2019). Moreover, the structure and regulation of the intestinal microbiota are emerging as diagnostic and therapeutic tools for patients with cancer. Despite mounting evidence suggesting close associations between the intestinal microbiome and NSCLC, research on the alteration of the intestinal microbiome and its metabolites, particularly SCFAs, in patients with NSCLC at various stages is limited. Additionally, the potential microbial biomarkers of NSCLC malignancy haven’t been adequately explored. Therefore, we conducted a study with 115 subjects, including 40 patients with ELC, 40 patients with BM of NSCLC, and 35 HCs, to examine biomarkers and the role of the gut microbiota in the progression of NSCLC. The findings may provide new insights into the involvement of the intestinal microbiome in the diagnosis and treatment of NSCLC, particularly in cases of BM.

Intestinal microbiome richness and evenness, as measured by three α-diversity indices, were found to be lower in patients with BM compared to ELC and HCs, consistent with prior studies (Liu et al., 2019). The loss of microbial ecology is well-documented in connection with chronic diseases (Budden et al., 2019). Firmicutes and Actinobacteria were less prevalent in the gut microbiota of patients with NSCLC, particularly in the BM stage. Firmicutes have been linked to immunological responses that regulate anti-inflammatory and apoptotic functions (Dalile et al., 2019). Actinobacteria, considered probiotics, has shown a reduction in colorectal cancer (Liu et al., 2017; Liu et al., 2019). Patients with NSCLC exhibited a lower ratio of Firmicutes to Bacteroidetes (F/B). A high F/B ratio typically indicates a healthy condition, and a reduced ratio may generate metabolic imbalance and decrease circulating SCFAs (Liu et al., 2019). This reduction in SCFAs was observed in the NSCLC group, especially in the BM stage, as measured by gas chromatography. SCFAs are known essential modulators of systemic inflammation and immunity, influencing intestinal and blood-brain barrier permeability (Dalile et al., 2019). They have demonstrated protective functions in various cancers by modulating programmed cell death and cell adhesion (Mirzaei et al., 2021).

Fusobacteria and Proteobacteria were more abundant in the gut microbiota of the NSCLC group, particularly in the BM group. Fusobacteria, known for opportunistic infections, may accelerate cancer progression by regulating T regulatory cells and activating autophagy (Liu et al., 2019). Proteobacteria, containing a large number of gram-negative pathogens (Vaz-Moreira et al., 2017), may imply intestinal epithelial barrier damage and susceptibility (Yoo et al., 2020). Variations at the family and genus levels were more complex. All evidence pointed toward a disruption in the balance of the intestinal microbiota in patients with NSCLC, particularly in cases of BM. We hypothesize that NSCLC creates an imbalanced intestinal microenvironment via the “gut–lung axis,” and this disrupted intestinal microbiome could potentially promote the progression of NSCLC. This assumption needs further validation through future animal research.

The potential ELC-associated intestinal microbiome revealed in this study sheds light on the intricate association between gut microbiota and the progression of NSCLC. The presence of the genus *Streptococcus*, known to promote tumor advancement by degrading tannic acid (Oehmcke-Hecht et al., 2020), suggests a potential contributing factor in early-stage NSCLC. On the contrary, *Blautia*, enriched in ELC, has been reported to have preventive benefits against carcinogenesis and probiotic qualities by promoting SCFA generation and suppressing inflammatory responses (Liu et al., 2021b). The transient compensating response hypothesis is considered, indicating a complex interplay of microorganisms in response to the early stages of NSCLC.

Four BM-associated microorganisms were identified, including *Enterobacteriaceae* and *Klebsiella*, both known for their potential to produce colicins that may damage DNA and contribute to the occurrence and progression of various cancers. *Enterobacteriaceae*’s association with an imbalanced immunological environment (Yoo et al., 2020) and its potential to alter the gut milieu suggest a compromised immunological and metabolic environment in individuals with BM of NSCLC. These findings highlight the connection between intestinal microbiome profiles and cancer risk. Low-abundance microorganisms in patients with NSCLC, such as *Faecalibacterium*, *Bifidobacterium*, and *Butyricicoccus*, were also revealed. *Faecalibacterium* and *Butyricicoccus*, known for their anti-tumor effects through butyric acid (Li et al., 2022) generation, play crucial roles in maintaining gut physiology (Singhal et al., 2021), influencing immunological responses, inhibiting pathogen invasion, and suppressing tumor development (Chen et al., 2020). Additionally, *Bifidobacterium*, a primary acetate source, effectively resists cancer by maintaining the dynamic equilibrium of metabolism and immunity (Badgeley et al., 2021). The decline of these SCFA-producing microorganisms may serve as a risk factor for the progression of NSCLC. The creation of a diagnostic model comprised of six abundant microorganisms for patients with NSCLC at various stages, with satisfactory predictive performance demonstrated by ROC curves, suggests the potential of the intestinal microbiome as a noninvasive diagnostic tool.

Surprisingly, the observed alterations in the gut microbiota of patients with ELC and BM suggest close associations between these stages. The deficiency of butyrate-producing bacteria, such as *Butyricicoccus* in Firmicutes, could promote the colonization of pathogenic *Enterobacteriaceae* (Proteobacteria) by upregulating intestinal pH (Yoo et al., 2020). The reciprocal association, where increasing *Enterobacteriaceae* inhibits SCFA-producing bacteria (Yoo et al., 2020), creates a potential vicious circle. Furthermore, the expansion of gram-negative bacteria like *Enterobacteriaceae* and *Klebsiella* may promote facultative anaerobic pathogens by creating lipopolysaccharide (Yoo et al., 2020), contributing to the drastic reduction of strict obligatory anaerobes of Firmicutes and Bifidobacteriaceae (Actinobacteria) due to the disruption of the intestinal oxygen environment. This holistic dysbiosis of the microbiome, rather than specific pathogens, may be tightly associated with the development of NSCLC. These findings highlight the intricate interactions within the gut microbiota and their potential impact on the progression of NSCLC.

The KEGG prediction pathways uncovered in this study provide insights into the complex interactions between gut microbiota and the host in patients with NSCLC. The disruption of various vital physiological processes, including lipid metabolism and genetic information processing, indicates a multifaceted impact on cancer progression. Despite the decrease in SCFAs, an increase in fatty acids, especially in BM patients, suggests a potential role of long fatty acids produced by *Enterobacteriaceae* (Mitchell and Ellermann, 2022). This could be significant, as fatty acid biosynthesis derived from acetyl-coenzyme A is critical for cancer cell signaling molecules and membrane biosynthesis. Furthermore, the disturbed pathways related to genetic information processing, such as DNA replication and mismatch repair, point to aberrant epigenetic processes in NSCLC.

The complexity of interactions between the intestinal microbiome and the host is highlighted, suggesting that NSCLC is not only a genetic but also a metabolic illness with concurrent anomalies in the intestinal microbiome. The microbiome may play a ruling role in the NSCLC system, impacting and being impacted by alterations in metabolism and genetic information. The study suggests the initiation of the process by carcinogenic metabolites or toxins that modulate metabolism and genetic information.

The potential influence of microbial community regulation by drugs on NSCLC development (Huang et al., 2022). and the enhancement of anti-cancer therapy efficacy through microbial community variation opens avenues for novel therapeutic approaches (Badgeley et al., 2021). Considering the potential superior clinical efficacy of targeted therapies, the suggestion to use probiotics or SCFAs to combat functional dysbiosis and even BM in chemoradiation-treated patients is noteworthy. This study, being the first to investigate the association between intestinal microbiota and its metabolites in ELC and patients with BM, provides fresh insights into the underlying roles of the microbiome in diagnosing and treating NSCLC.

However, the study acknowledges significant drawbacks, including a relatively small sample size, a broad grouping scope, and the absence of specific subtyping for ELC. The limitation of amplification bias in the 16S rRNA gene sequencing method is recognized. While the data suggest an association between dysbiosis of the intestinal microbiome and disruptions in metabolism and immunology in patients with NSCLC, the study emphasizes the need for more extensive and rigorous experiments to address these questions.

## Conclusions

In the context of NSCLC, this study revealed a notable lack of microbial diversity and SCFA-producing bacteria, particularly evident in patients with BM. Furthermore, the identification of a microbial biomarker panel consisting of *Faecalibacterium*, *Bifidobacterium*, *Butyricicoccus*, *Klebsiella*, *Streptococcus*, and *Blautia* presents a promising avenue for noninvasive diagnostic tools in patients with ELC and BM. The findings underscore the potential of gut microbiota-based biomarkers in the diagnostic landscape. The KEGG pathways analysis highlighted aberrations in multiple lipid metabolism-related and genetic information processing pathways in patients with NSCLC, especially those with BM. The substantial correlation observed between fecal microbiota and SCFAs with the malignant degree of NSCLC suggests a potential association between the reduction of SCFA-producing bacteria and the development and progression of NSCLC, including the process of BM. However, the study acknowledges the possibility that these microbial alterations may be a consequence of tumor progression. As a final note, the study emphasizes the need for more extensive and rigorous research in the future to delve deeper into the intricate association between gut microbiota, SCFAs, and the progression of NSCLC.

## Data availability statement

The datasets presented in this study can be found in online repositories. The names of the repository/repositories and accession number(s) can be found below: https://www.ncbi.nlm.nih.gov/, PRJNA957735.

## Ethics statement

The studies involving humans were approved by The Affiliated Hospital of Yangzhou University’s Ethics Committee. The studies were conducted in accordance with the local legislation and institutional requirements. The participants provided their written informed consent to participate in this study.

## Author contributions

DC, AP, and YL conceived and designed this project. YW, XZ, and WZ collected the samples and performed the data analysis. HJ, WZ, and XZ conducted the experiments and wrote the manuscript. All authors read and approved this manuscript.
