# Supplementary material for: Gut microbiota and its metabolites in non-small cell lung cancer and brain metastasis: from alteration to potential microbial markers and drug targets
Source: Front Cell Infect Microbiol. 2024 Jan 18;13:1211855. doi: 10.3389/fcimb.2023.1211855 (PMC10830900; doi:10.3389/fcimb.2023.1211855)
Supplement: Supplementary file 1 [file Table_1.docx]

**Supplementary Table 1. The relative abundance of** **[dominan](javascript:;)t gut microbiota among NSCLC, BM and HC groups.**

|  | mean_HC (n=40) | mean_ELC (n=40) | mean_BM (n=35) | *p* value | FDR-corrected *p* values |
| --- | --- | --- | --- | --- | --- |
| p_Firmicutes | 0.472213333 | 0.392953913 | 0.335159231 | 0.042 | 0.025 |
| p_Bacteroidetes | 0.4116 | 0.435718696 | 0.428913077 | 0.66 | 0.20 |
| p_Proteobacteria | 0.041405 | 0.095142174 | 0.134079231 | 0.035 | 0.025 |
| p_Actinobacteria | 0.031245385 | 0.012692174 | 0.006874167 | 0.023 | 0.025 |
| p_Fusobacteria | 0.012988333 | 0.035593478 | 0.063410769 | 0.018 | 0.025 |
| p_Verrucomicrobia | 0.011287917 | 0.016297696 | 0.017041538 | 0.25 | 0.11 |
| p_Cyanobacteria | 0.0031975 | 0.002707391 | 0.002933077 | 0.34 | 0.13 |
| p_Desulfobacterota | 0.002502213 | 0.002823102 | 0.002012011 | 0.63 | 0.19 |
| p_Tenericutes | 0.002333578 | 0.001267328 | 0.001566541 | 0.41 | 0.15 |
| p_Synergistetes | 0.001854122 | 0.001002136 | 0.001211766 | 0.57 | 0.18 |
| Firmicutes/Bacteroidetes | 1.147262714 | 0.901852311 | 0.781415277 | 0.029 | 0.025 |
| f_Bacteroidaceae | 0.255794583 | 0.275453913 | 0.267899231 | 0.52 | 0.13 |
| f_Lachnospiraceae | 0.202546667 | 0.144296957 | 0.103331538 | 0.016 | 0.012 |
| f_Prevotellaceae | 0.093594167 | 0.051598696 | 0.041387692 | 0.017 | 0.012 |
| f_Ruminococcaceae | 0.11986375 | 0.108783043 | 0.091576923 | 0.16 | 0.045 |
| f_Bifidobacteriaceae | 0.02867125 | 0.010236522 | 0.004846152 | 0.021 | 0.012 |
| f_Acidaminococcaceae | 0.03923913 | 0.036889583 | 0.014520769 | 0.038 | 0.014 |
| f_Enterobacteriaceae | 0.021622083 | 0.077663913 | 0.113223077 | 0.0070 | 0.012 |
| f_Veillonellaceae | 0.01083 | 0.014557391 | 0.013853846 | 0.049 | 0.015 |
| f_Oscillospiraceae | 0.020423333 | 0.024301304 | 0.016666923 | 0.55 | 0.13 |
| f_Streptococcaceae | 0.015643333 | 0.011416522 | 0.009304615 | 0.043 | 0.015 |
| g_Bacteroides | 0.255794583 | 0.275453913 | 0.267899231 | 0.52 | 0.12 |
| g_Prevotella_9 | 0.082546667 | 0.044296957 | 0.033331538 | 0.013 | 0.0080 |
| g_Faecalibacterium | 0.103594167 | 0.086598696 | 0.066387692 | 0.043 | 0.013 |
| g_Bifidobacterium | 0.02867115 | 0.010236413 | 0.004846023 | 0.020 | 0.0099 |
| g_Phascolarctobacterium | 0.038525416 | 0.034153478 | 0.01374 | 0.030 | 0.012 |
| g_Roseburia | 0.03128875 | 0.016190435 | 0.004151538 | 0.0018 | 0.0019 |
| g_Dorea | 0.009323333 | 0.00817913 | 0.005529231 | 0.064 | 0.017 |
| g_Escherichia/Shigella | 0.015131667 | 0.031056957 | 0.030140769 | 0.036 | 0.012 |
| g_Lachnospira | 0.020423333 | 0.014301304 | 0.016666923 | 0.24 | 0.060 |
| g_Klebsiella | 0.005012333 | 0.041416522 | 0.082304615 | 0.0018 | 0.0019 |

Abbreviations: HC, healthy control; ELC: an early stage of non-small cell lung cancer; BM: brain metastasis; p, phylum; f, family; g, genus.
